# Supplementary material for: The structural basis for dynamic DNA binding and bridging interactions which condense the bacterial centromere
Source: eLife. 2017 Dec 15;6:e28086. doi: 10.7554/eLife.28086 (PMC5731820; doi:10.7554/eLife.28086)
Supplement: Figure 3—source data 1. — acommonly assigned groups (i.e. excluding OH, Asp/Glu side-chain carbonyl, Lys amide and Arg guanidinium groups as well as tertiary aromatic carbons); bresidues 229–282; cvalues reported by ARIA 2.3 (Rieping et al., 2007); dordered residues (230-254, 259-278) as calculated by PSVS 1.5 (Bhattacharya et al., 2007); evalues reported by Procheck (Laskowski et al., 1993); fvalue reported by PDB validation software; g residues in secondary structure (231-245, 249-254, 257-264, 267-277). The structural validation programs used were as follows: CING (Doreleijers et al., 2012), Verify3D (Lüthy et al., 1992), Prosa II (Sippl, 1993), Procheck (Laskowski et al., 1993), MolProbity (Lovell et al., 2003). [file elife-28086-fig3-data1.docx]

| **Degree of assignment**^a,b^ |  |
| --- | --- |
| Backbone (Cα, C', N and H^N^) (%) | 98.5 |
| Side-chain H (%) | 97.5 |
| Side-chain non-H (%) | 96.2 |
| **Number of restraints** (per monomer) |  |
| NOE restraints |  |
| Intra-residue (\|i-j\| = 0) | 477 |
| Sequential (\|i-j\| = 1) | 319 |
| Medium range (2 ≤ \|i-j\| < 5) | 324 |
| Long range (\|i-j\| ≥ 5) | 392 |
| Ambiguous | 481 |
| Total | 1993 |
| H-bond restraints (intra-/intermonomer) | 24 / 4 |
| Dihedral angle restraints (/) | 40 / 40 |
| **Restraint statistics**^c^ |  |
| r.m.s. of NOE violations (Å) | 0.09 ± 0.01 |
| r.m.s. of H-bond violations (Å) | 0.19 ± 0.11 |
| r.m.s. of dihedral violations (°) | 0.15 ± 0.07 |
| **r.m.s. from idealised covalent geometry**^c^ |  |
| Bonds (Å) | 0.0032 ± 0.0001 |
| Angles (°) | 0.48 ± 0.015 |
| Impropers (°) | 1.20 ± 0.12 |
| **Structural quality** |  |
| Ramachandran statistics^e,b/d^ |  |
| Most favoured regions (%) | 87.4 / 93.6 |
| Allowed regions (%) | 10.8 / 6.4 |
| Generously allowed regions (%) | 0.4 / 0.0 |
| Disallowed regions (%) | 1.4 / 0.0 |
| CING (51) % ROG scores (R/O/G)^b^ | 19 / 15 / 67 |
| Verify3D (71) Z-score^b^ | -0.16 |
| Prosa II (72) Z-score^b^ | -1.78 |
| Procheck (73) Z-score (/)^b,d^ | 0.79 |
| Procheck (73) Z-score (all)^b,d^ | -0.35 |
| MolProbity (74) Z-score^b^ | -2.51 |
| No. of close contacts^b,f^ | 4 |
| **Coordinates precision (rmsd)** ^b/d/g^ |  |
| All backbone atoms (Å) | 0.8 / 0.5 / 0.4 |
| All heavy atoms (Å) | 1.0 / 0.8 / 0.7 |

**Figure 3-source data 1 – NMR assignment, structure calculation and validation statistics**
