# Supplementary material for: The structural basis for dynamic DNA binding and bridging interactions which condense the bacterial centromere
Source: eLife. 2017 Dec 15;6:e28086. doi: 10.7554/eLife.28086 (PMC5731820; doi:10.7554/eLife.28086)
Supplement: Transparent reporting form [file elife-28086-transrepform.pdf]

eLIFE
1st Floor
24 Hills Road
Cambridge CB2 1JP, UK
P 01223 855340
W elifesciences.org
T @elife

eLife's transparent reporting form

We encourage authors to provide detailed information *within their submission* to facilitate the interpretation and replication of experiments. If you have any questions, please contact us: editorial@elifesciences.org.

Sample-size estimation

- You should state whether an appropriate sample size was computed when the study was being designed
- You should state the statistical method of sample size computation and any required assumptions
- If no explicit power analysis was used, you should describe how you decided what sample (replicate) size (number) to use

Please outline where this information can be found within the submission (e.g., page numbers or figure legends), or explain why this information doesn't apply to your submission:

No power analysis was used to prepare statistical samples. This is not relevant to molecular structure determination by NMR or to analysis using the other biophysical techniques used herein, where technical and biological reproducibility is the primary concern.

Replicates

- You should report how often each experiment was performed
- You should include a definition of biological versus technical replication
- The data obtained should be provided and sufficient information should be provided to indicate the number of independent biological and/or technical replicates
- If you encountered any outliers, you should describe how these were handled
- Criteria for exclusion/inclusion of data should be clearly stated
- High-throughput sequence data should be uploaded before submission, with a private link for reviewers provided (these are available from both GEO and ArrayExpress)

Please outline where this information can be found within the submission (e.g., page numbers or figure legends), or explain why this information doesn't apply to your submission:

eLife Sciences Publications, Ltd is a limited liability non-profit non-stock corporation incorporated in the State of Delaware, USA, with company number 5030732, and is registered in the UK with company number FC030576 and branch number BR015634 at the address 1st Floor, 24 Hills Road, Cambridge CB2 1JP | August 2014

Statement of the number of experiment replicates undertaken:
- NMR structure determination and protein preparation – n/a
- CD spectroscopy – page 19
- EMSA analysis – page 21
- PIFE assay – page 22
- Magnetic tweezers – Figure 2, as an example
- Native mass spectrometry – page 25
- AUC – page 25
- in vivo fluorescence imaging – page 26
- ChIP-qPCR – page 28
- Marker frequency analysis – page 29

Definition of biological vs technical repeats – page 25

The exclusion of outliers was not required in this study.

Statistical reporting

- Statistical analysis methods should be described and justified
- Raw data should be presented in figures whenever informative to do so (typically when N per group is less than 10)
- For each experiment, you should identify the statistical tests used, exact values of N, definitions of center, methods of multiple test correction, and dispersion and precision measures (e.g., mean, median, SD, SEM, confidence intervals; and, for the major substantive results, a measure of effect size (e.g., Pearson's r, Cohen's d)
- Report exact p-values wherever possible alongside the summary statistics and 95% confidence intervals. These should be reported for all key questions and not only when the p-value is less than 0.05.

Please outline where this information can be found within the submission (e.g., page numbers or figure legends), or explain why this information doesn't apply to your submission:

Dispersion and precision measures were used in this study. Details for these are included in the appropriate Figure legends and stated within the materials and methods section:<br>- Figure 2 (legend, page 31)<br>- Figure 5 (legend, page 34)<br>- Figure 6 (legend, page 35)<br>- Figure 2 – Figure Supplement 1 (legend, page 46)<br>- Figure 4 – Figure Supplement 1 (legend, page 51)<br>- Figure 5 – Figure Supplement 1 (legend, page 52)<br>- Materials and methods, pages 22, 25, 29<br><br>Where in-built software functions were applied, the name of the software and function used is stated.<br>- Figure 3 – source data 1 (legend, page 49)<br>- Materials and methods, pages 21, 22 and 25<br><br>Where appropriate, both raw data and bulk analysis are included e.g. in magnetic tweezer studies, raw data was resampled from 120 Hz (acquisition sampling rate) to 2 Hz for both representation and analysis (page 22). For ChIP-qPCR analysis, raw data was considered more transparent and rich and therefore preferable to using normalized data representation (Figure 7, page 36).

(For large datasets, or papers with a very large number of statistical tests, you may upload a single table file with tests, Ns, etc., with reference to page numbers in the manuscript.)

Additional data files ("source data")

- We encourage you to upload relevant additional data files, such as numerical data that are represented as a graph in a figure, or as a summary table
- Where provided, these should be in the most useful format, and they can be uploaded as "Source data" files linked to a main figure or table
- Include model definition files including the full list of parameters used
- Include code used for data analysis (e.g., R, MatLab)
- Avoid stating that data files are "available upon request"

Please indicate the figures or tables for which source data files have been provided:

Figure 3 – source data 1 (NMR assignment, structure calculation and validation statistics)<br><br>The PDB validation report has also been uploaded with the submission.
